# The mediating role of emotion regulation strategies in the relationship between family of origin violence and intimate partner violence

**DOI:** 10.1186/s41155-021-00187-8

**Published:** 2021-07-29

**Authors:** Arturo Enrique Orozco-Vargas, Arturo Venebra-Muñoz, Ulises Aguilera-Reyes, Georgina Isabel García-López

**Affiliations:** 1 Universidad Autónoma del Estado de México, Toluca, México; 2 Centro Universitario UAEM Atlacomulco, Carretera Toluca - Atlacomulco Km. 60, Atlacomulco, México

**Keywords:** Emotion regulation strategies, Intimate partner violence, Family of origin violence, Mediating effect, Structural equation modeling

## Abstract

The effects of family of origin violence and intimate partner violence have been extensively documented; however, very few studies have examined the interaction with emotion regulation strategies. Thus, the objective of this research was to analyze whether different types of emotion regulation strategies, both adaptive and maladaptive, mediate the relationship between family of origin violence and intimate partner violence in the Mexican population. A total of 838 participants (45.9% men and 54.1% women) responded to instruments addressing family of origin violence, emotion regulation strategies, and intimate partner violence. The results revealed that both structural models were significant. For women, the model showed an adequate fit *X*^2^ (11, *N* = 838) = 22.75, *p* = .288, GFI = .95, AGFI = .91, NFI = .98, CFI = .97, RMSEA = .05. Likewise, we found similar indexes for men *X*^2^ (11, *N* = 838) = 28.20, *p* = .348, GFI = .97, AGFI = .93, NFI = .97, CFI = .95, RMSEA = .04. Specifically, the direct effects of adaptive strategies on intimate partner violence were statistically significant. Meanwhile, the direct effects of family of origin violence on maladaptive emotion regulation strategies were significant, as were the direct effects of maladaptive strategies on intimate partner violence. In turn, the indirect effects of family of-origin violence were significantly related to intimate partner violence via maladaptive emotion regulation strategies. In addition, the results clearly showed that men reported higher levels of aggression against women. Finally, regarding the selection of emotion regulation strategies, while women employed more adaptive emotion regulation, men showed a more definite tendency to use maladaptive emotion regulation.

## Introduction

Intimate partner violence (IPV) is one of the main risk factors in Mexican society. In the past two decades, and especially in the months of lockdown as a result of the COVID-19 pandemic, the prevalence of IPV has increased. Although the study of IPV has always been essential in many countries, its social relevance is more important in these days in Mexico. Different studies have documented the high prevalence of IPV in Mexico. One of the most recent studies revealed that 39.1% of participants reported low physical and sexual violence, 9.6% high sexual and low physical violence, 36.5% high physical and low sexual violence and injuries, and 14.8% of participants expressed having experienced high physical and sexual violence and injuries (Gupta et al., 2018).

Similarly, the most recent national survey conducted in Mexico (Encuesta Nacional sobre la Dinámica de las Relaciones en los Hogares [ENDIREH], National Survey on the Dynamics of Domestic Relationships), which included women aged 15 and older, revealed that 43.9% of them reported having been victims of an episode of violence perpetrated by their romantic partner at some point in their relationship. Specifically, 40.1% of Mexican females indicated having suffered emotion violence, characterized by intimidation (24.8%), indifference from their intimate partner (29.5%), and psychological degradation (22.1%). Similarly, 17.9% of the victims claimed to have suffered physical violence, manifested by slaps in 11.3% of cases, blows with the fist or some object in 8.1%, and hair pulling or pushing in 13.8%. At the same time, 6.5% of the victims have suffered sexual violence, with rape accounting for 5.5% and sexual abuse for 4.0%. Likewise, a type of violence that has grown in recent years is financial or patrimonial violence, which amounts to 20.9%; specifically, 17.4% of the victims mentioned having been blackmailed or controlled in terms of access to financial resources, and 14.9% of them reported they were abused for not having fulfilled their economic responsibilities. Regarding female teenagers, 40.3% of them reported they had been abused by their intimate partner at some point in the relationship. In indigenous female teenagers, this percentage increases to 46.1% (Instituto Nacional de Estadística y Geografía [INEGI], National Institute of Statistics and Geography, , 2017). Based on these data showing the high prevalence of IPV, it is necessary to design more studies in order to identify the causes of this type of violence. As a consequence, it will be possible to develop more preventive and intervention programs for victims and their families.

In the scientific literature, there has been an emphasis on the study of women as victims of IPV, with men as perpetrators. However, due to the increase in the number of violent acts committed by women and reports of victimization by men, investigations of intimate partner violence are turning increasingly two-way. Therefore, results do not make it clear whether it is men or women who exercise more violence against the other. Similarly, it is difficult to determine whether men or women are primarily the victims of partner violence. Particularly in Mexico, few studies have compared the prevalence of perpetration and victimization in men and women. Consequently, this study will contribute to fill this gap in the literature. Among the few that do stand out, there is a study carried out with the participation of college students living in northwestern Mexico. The results showed that women reported experiencing higher levels of physical violence as compared to men. However, regarding sexual violence, the reverse trend was seen, with more men reporting this kind of violence (Kaldman, Pérez, Rodríguez, & Valdez, 2020).

Meanwhile, family of origin violence (FOV) is also highly prevalent in Mexico. FOV has direct and indirect effects on Mexican men and, mainly, on Mexican women. The results of the most recent national survey carried out in Mexico (ENDIREH, 2016) revealed that, in general, 8.1% of female participants mentioned having frequently suffered various episodes of direct psychological and/or physical FOV. Likewise, 12.3% indicated having experienced indirect psychological and/or physical FOV. Specifically, these acts of violence included having witnessed beatings among family members (24.9%), having witnessed insults or offenses in their family of origin (31.1%), having received beatings from people in the same household (32.1%), and having been insulted or offended (18.0%) (INEGI, 2017).

Due to the wide variety of effects that FOV has in later stages of development (Casique, 2018), we have sought to determine its incidence in IPV. Several studies have documented the impact of having witnessed or directly suffered different types of psychological, physical, and sexual abuse by parents, siblings, or other family members when the victim was a child or teenager (e.g., Eriksson & Mazerolle, 2015; Frías & Erviti, 2014; Rivas, Bonilla, & Vázquez, 2020; Smith‐Marek et al., 2015). In this intergenerational transmission of violence, it has been estimated that having been exposed to several episodes of FOV increases the probability to exert violence in an intimate relationship by 2–4 times (Iverson, Jimenez, Harrington, & Resick, 2011). Specifically, having experienced violence in the family of origin impacts both the perpetrator’s and victim’s intimate partner relationships by developing attitudes that ingrain the use of violence (Carr & VanDeusen, 2002), leads them to show serious deficiencies in the regulation of their emotions (Katz, Stettler, & Gurtovenko, 2015), and results in their presenting different psychological disorders, mainly anxiety, depression, hostility, and stress (Dardis, Dixon, Edwards, & Turchik, 2015; Machisa, Christofides, & Jewkes, 2016; Norman et al., 2012). Although the effects of FOV have been identified in many international studies, there is a lack of research addressing the academic and social impact in Mexico. Therefore, the relevance of this study is based on the new contributions that we will provide.

It has been documented that FOV and IPV affect not only the victim, but also their immediate environment, mainly their children. Finding a comprehensive explanation to elucidate the origin and development of these two types of violence has been a task undertaken by hundreds of researchers around the world. One of the areas of psychology that has recently shown interest in the study of IPV is emotion regulation. This construct is defined as the internal processes that a person carries out to maintain, inhibit, or increase emotion expressions and experiences (Gross, 2002). Emotion regulation allows managing and controlling the negative emotions that arise from unpleasant experiences. However, various individual circumstances and the complexity of the experience may have an impact on the ability to handle the arising emotions. The latter has been called emotion dysregulation, which refers to a person’s inability to have an adequate response to the emotions experienced, regardless of their nature or attributes. These maladaptive responses include difficulties in controlling the behavior triggered by distressing emotions, as well as deficits in the functional use of emotions (Gratz & Roemer, 2004).

According to the Process Model of Emotion Regulation proposed by Gross (1999), the process of emotion regulation can occur before or after the emotion response occurs. In this way, *antecedent*-*focused emotion* regulation occurs when a person anticipates an emotion response; for example, avoiding going to a party because she/he knows an ex with whom the relationship ended on very bad terms will be attending. Conversely, *response*-*focused emotion* regulation occurs after an emotion-charged event, such as apologizing to a sister for being angry and insulting her after learning she lost a valuable object (Gross, 1998).

Emotion regulation makes it possible to manage emotions considered as positive or, on the contrary, to control emotions of a negative nature. In order to describe an individual’s success or failure in controlling their emotions, two terms were proposed: adaptive emotion regulation and maladaptive emotion regulation (Gross, 2002). Adaptive emotion regulation refers to the process related to the control of emotions that allows a person to function successfully in his/her environment (Bridges, Denham, & Ganiban, 2004). Thus, when faced with a situation involving mainly negative emotions, emotion regulation leads the individual to use a series of strategies and skills that will allow him/her not only to cope with that situation, but in many cases to resolve it successfully. Among the main strategies of adaptive emotion regulation are mindfulness, reappraisal, acceptance, focusing, and problem solving. On the contrary, maladaptive emotion regulation occurs when an individual is unable to handle a situation that arouses negative emotions and to redirect it toward the achievement of his/her goals. Sometimes, the lack of regulatory skills prevents the individual from following the appropriate course of action in the face of the emotion experience (Aldao, Nolen-Hoeksema, & Schweizer, 2010). The main strategies of maladaptive emotion regulation include rumination, suppression, avoidance, difficulties in impulse control, and limited access to regulation strategies.

The study of emotion regulation in victims of FOV and victims of IPV is not yet considerably studied. Only some few studies have analyzed the effects of FOV in emotion regulation strategies. In a research analyzing mother-child dyads, results showed that children’s emotion dysregulation was associated with episodes of intimate violence perpetrated by mothers and their partner (Harding, Morelen, Thomassin, Bradbury, & Shaffer, 2013). Similarly, in another study including children who lived in a family environment characterized by high levels of violence, results showed that children who experienced or witnessed more severe violence between their parents reported worse emotion regulation skills (Howell, Graham-Bermann, Czyz, & Lilly, 2010). A general conclusion of these and other studies is that exposure to FOV has a severe and enduring impact in the capacity for managing emotions. For this reason, the analysis of this relation is highly relevant because of its social and academic consequences.

On the other hand, deficient emotion regulation strategies have also negative effects in the lives of victims and perpetrators. Due to the incapacity to deal with emotionally problematic situations as well as the presence of maladaptive coping skills in relational settings, emotion dysregulation is considered a risk factor for many couples who experience abuse and violence in their romantic relationships. Several studies have documented the relation between emotion regulation and IPV showing how the incompetence to control emotions may affect romantic interactions among couples. For instance, when partners report higher levels of emotional dysregulation, they are more prone to experience sexual and physical violence perpetration (Lee, Rodriguez, Edwards, & Neal, 2020). Similarly, emotional clarity and difficulties in the control of impulses, two of the most important emotion regulation strategies, were related to psychological violence in male perpetrators (Bliton et al., 2016). In turn, among female and male young adults, deficits in the expression and modulation of anger-related dysregulation are associated with physical and psychological IPV victimization (Iverson, McLaughlin, Adair, & Monson, 2014). In previous research, it was identified that some individuals tend to be involved romantically with others who present similar emotion regulations strategies. When both partners present high levels of anger-related dysregulation, this condition would increase the risk of IPV victimization (Segrin, 2004).

Although some studies have begun to address the impact of some emotion regulations strategies in IPV; still, it is necessary to investigate why some strategies have more effects than others in the prevalence of IPV and what strategies are more used by perpetrators and victims. These objectives show the social and academic relevance of continuing with the development of more studies analyzing this relation.

**Fig. 1:**
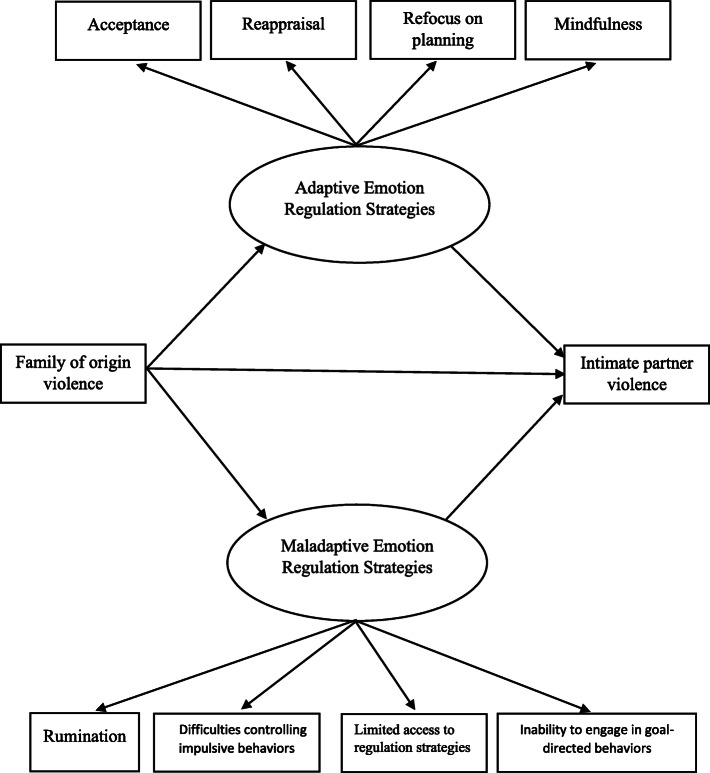
Hypothesized measurement model

Based on the effects that adaptive and maladaptive emotion regulation strategies have, we propose a model in which family of origin violence impacts both types of strategies, which in turn increase the possibility of developing high levels of intimate partner violence (see Fig. 1).

After an exhaustive review of the literature, we did not find any research that examined the effects of emotion regulation strategies on intimate partner violence using a structural equation model. In order to fill this gap in the scientific literature, the general objective of this research was to analyze whether different types of emotion regulation strategies, both adaptive (mindfulness, acceptance, positive reappraisal, and refocus on planning) and maladaptive (rumination, impulse control difficulties, limited access to regulation strategies, and interference in targeted behaviors) mediate the relationship between family of origin violence and intimate partner violence in the Mexican population. Specifically, the hypothesis was that maladaptive emotion regulation strategies may have a greater impact on intimate partner violence. We also analyzed the correlation between adaptive and maladaptive emotion regulation strategies and physical, psychological, and sexual partner violence in males and females from a non-clinical sample. Thus, another hypothesis was that men may have more difficulty in controlling their emotions than women. At the same time, we sought to determine whether the three types of partner violence were related primarily to maladaptive emotion regulation strategies.

## Method

### Design and participants

This is a cross-sectional, correlational, and predictive design with a structural equation model (Tabachnick & Fidell, 2012). The sample consisted of 838 participants, 385 men (45.9%) and 453 women (54.1%). The participants were 18–57 years old (*M* = 32.57, *SD* = 8.49) and lived in Mexico’s central zone. The inclusion criterion was to be a member of a romantic couple living together. They reported to have lived with their intimate partner for less than one year (0.48%), 1–5 years (12.04%), 6–10 years (28.79%), 11–15 years (20.70%), 16–20 years (11.73%), 21–25 years (13.78%), 26–30 years (8.14%), or over 35 years (4.43%). In terms of marriage, 73.84% of the participants were married and 26.16% were cohabiting. Concerning the number of children, 12.42% had no children, 18.43% had one child, 44.24% had two children, 14.89% had three children, 7.98% had four children, and 2.04% had five or more children. Most of the participants had finished high school (60.82%), followed by those who had only finished junior high (15.72%), and those with a bachelor's degree (13.24%); a smaller percentage mentioned that they had only finished elementary school (7.28%), or a graduate degree (2.94%).

### Instruments

The Cognitive Emotion Regulation Questionnaire (CERQ) created by Garnefski, Kraaij, and Spinhoven (2001) includes 36 items grouped into nine factors (Rumination, Catastrophizing, Self-blame, Positive Reappraisal, Refocus on planning, Acceptance, Positive refocusing, Other-blame, and Putting into Perspective). The objective of this instrument is to measure the general cognitive style, as well as the emotion strategies that are used after having lived a very particular event. Based on the purpose of this research, only the dimensions of Rumination, Acceptance, Positive Reappraisal, and Refocus on planning were included. Rumination measures excessive thinking about the feelings and ideas that arise because of an unpleasant event (e.g., I often think about how I feel about what I have experienced). Acceptance refers to the thoughts through which it is accepted that an unpleasant event occurred (e.g., I think that I have to accept that this has happened). Positive reappraisal measures the thoughts that are generated with the aim of giving positive meaning to events considered unpleasant (e.g., I think that I can become a stronger person as a result of what has happened). Finally, refocus on planning involves thinking about all the steps that must be followed to achieve the solution of the problems that are being experienced (e.g., I think about how I can best cope with the situation). Among the Spanish versions of the CERQ, in this research we used the proposal suggested by Domínguez-Sánchez, Lasa-Aristu, Amor, and Holgado-Tello (2013). The instrument has five response options in a Likert-type format ranging from 1 (almost never) to 5 (almost always). The CERQ has been used both in the general population and in the clinical setting reporting very adequate psychometric properties. Regarding internal consistency, coefficients between .68 and .93 have been identified. In the same way, convergent and discriminant validity has been analyzed through the correlations between the CERQ dimensions and other variables such as depression and anxiety, finding good indicators (Garnefski & Kraaij, 2007). In this research, we found an adequate internal consistency for rumination (Chronbach's alpha .81), acceptance (Chronbach’s alpha .72), positive reappraisal (Chronbach's alpha .88), and refocus on planning (Chronbach's alpha .84).

The Difficulties in Emotion Regulation Scale (DERS) was designed by Gratz and Roemer (2004) and consists of 36 items grouped into six dimensions: The first dimension, Difficulties controlling impulsive behaviors has six items; the second dimension, Limited access to regulation strategies contains eight items; the next dimension, Nonacceptance of negative emotions includes six items; the fourth dimension, Inability to engage in goal-directed behaviors has five items; the fifth dimension, Lack of emotion awareness has six items; and the last dimension, Lack of emotion clarity contains five items. In the present research, only the dimensions of Difficulties controlling impulsive behaviors, Limited access to regulation strategies, and Inability to engage in goal-directed behaviors were included. The Difficulties controlling impulse dimension measures the problems a person faces when trying to control the effects of negative emotions (e.g., When I’m upset, I lose control over my behavior). In turn, Limited access to regulation strategies refers to the difficulties that arise in identifying the skills necessary to manage emotions, mainly negative ones (e.g., When I’m upset, I believe there is nothing I can do to feel better). Finally, Inability to engage in goal-directed behaviors refers to the blockage generated by negative emotions causing a lack of concentration as well as the inability to carry out actions aimed at achieving personal goals (e.g., When I’m upset, I have difficulty getting work done). The DERS has an emphasis on measuring negative emotions because it assesses the difficulties people have in regulating their emotions. Due to the nature of this scale, the higher scores reflect the level of emotion dysregulation. In order to analyze this construct, the Spanish version proposed by Medrano, Moretti, Ortiz, and Pereno (2013) was used. The scale presents a Likert-type format with 5 response options that range from 1 (almost never) to 5 (almost always). The psychometric properties have been very adequate with an internal consistency of the total score of α = .94, as well as for the six dimensions which have oscillated between α = .80 and α = .91). For the dimensions included in this study, we found an internal consistency of .88 for Difficulties controlling impulsive behaviors, .82 for Limited access to regulation strategies, and .77 for Inability to engage in goal-directed behaviors.

The Five Facets Mindfulness Questionnaire (FFMQ) developed by Baer, Smith, Hopkins, Krietemeyer, and Toney (2006) includes 39 items. It measures five abilities inherent to mindfulness (Observation, Lack of judgment, Lack of reaction, Description of the experience and Conscious action) (e.g., I find it difficult to stay focused on what’s happening in the present moment; When I feel something in my body, it’s hard for me to find the right words to describe it; When I have distressing thoughts or images, I feel calm soon after). The items are answered on a Likert-type scale from 1 (never) to 5 (always). In the present research, we use the Spanish version of this questionnaire translated and adapted by Cebolla et al. Cebolla et al. (2012). In its Spanish version, the questionnaire reported adequate internal consistency indices in its five dimensions (Cronbach’s alpha equal to or greater than .80), as well as appropriate psychometric properties of factor validity and external validity (Cebolla et al., 2012). The internal consistency in this study was α = .85.

The Violence Scale and Severity Index (La Escala de Violencia e Índice de Severidad) was created by Valdez-Santiago et al. (2006) with the purpose of measuring intimate partner violence. The scale consists of 27 items grouped into four dimensions (Psychological violence containing of 8 items, Physical violence including 7 items, Severe physical violence involving of 9 items, and Sexual violence including 3 items). The response options are presented in a Likert-type scale with 4 options (0 = never, 1 = sometime, 2 = several times, and 3 = many times). This scale measures the frequency of violent episodes that occur against the participant during the last 12 months. However, because the objective of this research was to analyze the perpetration of intimate partner violence, the structure of the items was modified. Therefore, instead of asking: Does your partner belittle or humiliate you in front of other people? The item was written as: Do you belittle or humiliate your partner in front of other people? Instead of asking: Have your partner used physical force to have sex with you? The question was: Do you use physical force to make your partner have sex with you? Instead of asking: Have your partner pushed you on purpose?, the item was written as: Have you pushed your partner on purpose ?, etc. The scale reports good psychometric properties with a Cronbach’s alpha of .99 that evaluated internal consistency. In addition, the factor analysis determined that the four factors explained 62.3% of the total variance (Valdez-Santiago et al., 2006). In this study, Cronbach’s alpha internal consistency coefficients for physical violence were .82, for severe physical violence were .79, for psychological violence were .94, and for sexual violence were .87.

The Revised Conflicts Tactics Scale (CTS2) was created by Straus, Hamby, Boney-McCoy, and Sugarman (1996) to measure the perpetration of physical, psychological, and sexual violence committed by both partners. The scale includes 78 items (39 for each member of the couple) grouped into five dimensions (physical violence, psychological violence, sexual violence, negotiation, and injuries). In the present research, only physical violence (24 items), psychological violence (16 items), and sexual violence (14 items) factors were included. Participants were instructed to point out the acts of violence that their father perpetrated against their mother, as well as their mother against their father during the year in which they remember there were more conflicts between them. Thus, the participants reported acts of aggression and victimization on the psychological violence subscale (e.g., my father insulted or cursed my mother), on the physical violence subscale (e.g., my mother slapped my father), and on the sexual violence subscale (e.g., my father threatened my mother to make her have sex with him). Participants respond in a Likert-type format ranging from 0 (never occurred) to 6 (occurred more than 20 times). The CTS2 is one of the most used tests in the world reporting very acceptable psychometric properties. In addition to having an appropriate construct validity, its internal consistency is very adequate, ranging between .79 and .95 (Straus et al., 1996). Internal reliability in the current study for physical violence was .88, for psychological violence was .93, and for sexual violence was .82.

### Procedure

In order to carry out this research, 15 health institutions were initially selected for data collection to ensure the heterogeneity of the sample. After talking with the authorities in each of them, authorization was obtained from four public and three private health institutions. The inclusion of private and public institutions had the purpose of balance the socioeconomic and education level of participants. Data collection was carried out in waiting rooms. Potential participants were asked individually if they would agree to answer a series of psychological instruments. Those who agreed were given an informed consent form and an explanation of their rights as participants, the data collection procedure, and the objectives of the study. Participants understood that they could leave the study at any time if they wished to. Once they agreed to participate, they signed the informed consent form. They were asked to sit in a place away from the rest of the people who were waiting, for the sake of privacy and confidentiality in their answers. Besides the psychological scales, participants answered a sociodemographic questionnaire. An investigator was with them at all times to answer any questions they might have. Therefore, all participants completed the whole scales, and nobody was excluded due to missing data. The entire data collection process followed the guidelines and ethical principles implemented in Mexico for the development of research with human beings. None of the participants received financial compensation for answering the instruments. The average time it took them to complete all the tests was 20 min.

### Data analysis

After carrying out several descriptive analyses, a structural equation model was designed. This model includes a structural model describing potential causal associations between exogenous and endogenous variables and a measurement model depicting dependencies between latent variables and their indicators (Hoyle, 1995). Following the recommendations of Kline (2005), models consisting of two observed variables (family of origin violence and intimate partner violence) and two latent variables were analyzed. On the one hand, the latent variable of adaptive emotion regulation strategies was made up of four indicators: mindfulness, acceptance, positive reappraisal, and focus on plans. On the other hand, the maladaptive emotion regulation strategies consisted of four indicators: rumination, difficulties in impulse control, limited access to regulation strategies, and interference in goal-directed behaviors. Following the suggestion of Tabachnick and Fidell (2012) who proposed that a sample size of about 200 is necessary, we included 385 male participants and 453 female participants.

Using the statistical package SPSS, version 25.0, and the LISREL software, version 9.2 (Jöreskog, Sörbom, Du Toit, & Du Toit, 1999), several statistical analyses were carried out in order to confirm whether the data supported the theoretical model proposed in this research. According to Bentler (1986), one of the most important objectives in SEM modeling is to select, among a set of theoretically possible models, the best of these potential models. This final model must be designed based on theoretical rationales proposed in previous studies with the purpose of determining how well this model fits the theory. Therefore, it will be possible to confirm that the significant data have been modeled.

Following the recommendations of Hu and Bentler (1999), and Schumacker and Lomax (2016), the following goodness-of-fit indexes were included: the Chi-square statistic, the Normalized Fit Index (NFI), the Comparative Fit Index (CFI), the Goodness-of-Fit Index (GFI), the Adjusted Goodness-of-Fit Index (AGFI), and the root mean square error of approximation (RMSEA). A good fit of the proposed model is achieved if the values are equal to or greater than .95 for the NFI, CFI, GFI, values greater than .90 and close to .95 for AGFI, and close to .06 for the RMSEA.

The data collected were analyzed to examine the assumption of normality as well as to identify missing values and values outside of the scales. Only two participants were eliminated because there were several missing values and the presence of outlier values. In the rest of our statistical data, missing and outlier values were not found. Besides, evaluation of normality showed that all the dimensions were into the range of kurtosis and skewness of ± 2, confirming the validity of the normality assumption.

## Results

Initially, a series of preliminary analyses were carried out in order to evaluate the mean and standard deviation of each of the dimensions of the various instruments (see Table 1). This analysis was conducted separately for men and women. When examining means by sex, we found significant differences for women and men in acceptance, *t*(836) = 2.30, *p* < .01; mindfulness, *t*(836) = − 2.83, *p* < .01; rumination, *t*(836) = − 3.01, *p* < .01; limited access to emotion regulation strategies, *t*(836) = *3*.28, *p* < .01; interference in goal-directed behaviors, *t*(836) = 2.58, *p* < .01; physical family of origin violence, *t*(836) = 4.29, *p* < .01; psychological family of origin violence, *t*(836) = − 3.27, *p* < .01; sexual family of origin violence, *t*(836) = 4.50, *p* < .01; physical intimate partner violence, *t*(836) = 4.38, *p* < .01; severe physical intimate partner violence, *t*(836) = 3.72, *p* < .01; sexual intimate partner violence, *t*(836) = 5.18, *p* < .01.

**Table 1:** Means and standard deviations of study variables by sex

Variable	Women		Men	
	M	SD	M	SD
Adaptive emotion regulation strategies				
Acceptance	7.30	3.83	7.79	3.30
Reappraisal	13.72	3.23	13.59	3.38
Refocus on planning	8.47	4.18	8.53	3.53
Mindfulness	103.54	25.52	89.28	22.58
Maladaptive emotion regulation strategies				
Rumination	12.24	5.92	10.31	5.14
Difficulties controlling impulsive behaviors	15.22	4.82	15.33	5.72
Limited access to regulation strategies	15.83	5.44	20.13	6.24
Inability to engage in goal-directed behaviors	9.12	3.52	12.53	3.74
Family of origin violence				
Physical violence	52.78	16.41	59.13	14.73
Psychological violence	48.28	14.62	43.27	14.20
Sexual violence	21.48	9.37	25.58	12.49
Intimate partner violence				
Physical violence	6.07	2.56	9.92	3.01
Severe physical violence	10.63	3.51	16.36	4.17
Psychological violence	15.53	4.26	15.38	4.40
Sexual violence	2.12	1.61	5.27	3.25

Subsequently, a correlation analysis was carried out to evaluate the correlation between the study variables in men and women (see Table 2). Regarding the higher associations between family of origin violence and intimate partner violence, and emotion regulation strategies, the results showed that, for the group of women, higher associations were observed between intimate partner violence and rumination (*r* = .31, *p* < .01), between family of origin violence and focusing (*r* = − .25, *p* < .01), and between intimate partner violence and focusing (*r* = − *.*23, *p* < .01). On the other hand, in the group of males, the higher associations were identified between difficulties in impulse control and family of origin violence (*r* = − .27, *p* < .01), between limited access to emotion regulation strategies and intimate partner violence (*r* = 29, *p* < .01), and between difficulties in impulse control and intimate partner violence (*r* = .*26*, *p* < .01).

**Table 2:** Correlations of study variables for women and men

Variables	1	2	3	4	5	6	7	8	9	10
1.FOV	–	− .13**	− .07	− .25**	− .04	.14**	.10**	.09**	.06	.18**
2.Acceptance	− .10**	–	.42**	.38**	.53**	− .38**	− .34**	− .48**	− .35**	− .17**
3.Reappraisal	− .14**	.27**	–	.52**	.59**	− .42**	− .45**	− .50**	− .28**	− .15**
4.Refocus	− .18**	.22**	.34**	–	.55**	− .40**	− .51**	− .53**	− .34**	− .23**
5.Mindfulness	− .13**	.51**	.42**	.58**	–	− .25**	− .55**	− .48**	− .26**	− .19**
6.Rumination	.07	− .49**	− .55**	− .34**	− .48**	–	.49**	.52**	.38**	.31**
7.Difficulties	.27**	− .25**	− .40**	− .45**	− .53**	.35**	–	.56**	.41**	.13**
8.Limited access	.19**	− .62**	− .38**	− .52**	− .62**	.53**	.59**	–	.37**	.07*
9.Inability	.18*	− .18*	− .44**	− .47**	− .50**	.32**	.31**	.28**	–	.04
10.Intimate partner violence	.21**	− .15**	− .19**	− .18**	− .21**	.17**	.26**	.29**	.08	–

Correlations for women are showed on the top of the diagonal and correlations for men are displayed on the bottom half

*FOV* family of origin violence

*p < 0.05; **p < 0.01

The pattern of results for the correlation between emotion regulation strategies and FOV shows that, for both groups, there was a greater association between emotion regulation strategies and IPV as compared to the association between emotion regulation strategies and FOV. However, specifically in the group of male participants, a higher correlation was identified between maladaptive emotion regulation strategies and FOV, as well as between maladaptive emotion regulation strategies and IPV when compared to the group of females.

In a third step, a confirmatory factor analysis was carried out to examine the overall objective of this research. To this end, a structural model was designed to determine its validity as a hypothesized measurement model (see Fig. 1). When tested, the results showed that only some of the adjustment rates for the initial model were acceptable for both women *X*^2^ (12, *N* = 838) = 23.75, *p =* .044, GFI = .84, AGFI = .80, NFI = .92, IFC = .93, RMSEA = .05 and men, *X*^2^ (12, *N* = 838) = 31.09, *p =* .027, GFI = .88, AGFI = .85, NFI = .79, CFI = .93, RMSEA = .11. The direct effects of family of origin violence on adaptive emotion regulation strategies were not significant; however, the direct effects of adaptive strategies on intimate partner violence were statistically significant. Meanwhile, the direct effects of family of origin violence on maladaptive emotion regulation strategies were significant, as were the direct effects of maladaptive strategies on intimate partner violence. In turn, the indirect effects of family of-origin violence were significantly related to intimate partner violence via maladaptive emotion regulation strategies.

**Fig. 2:**
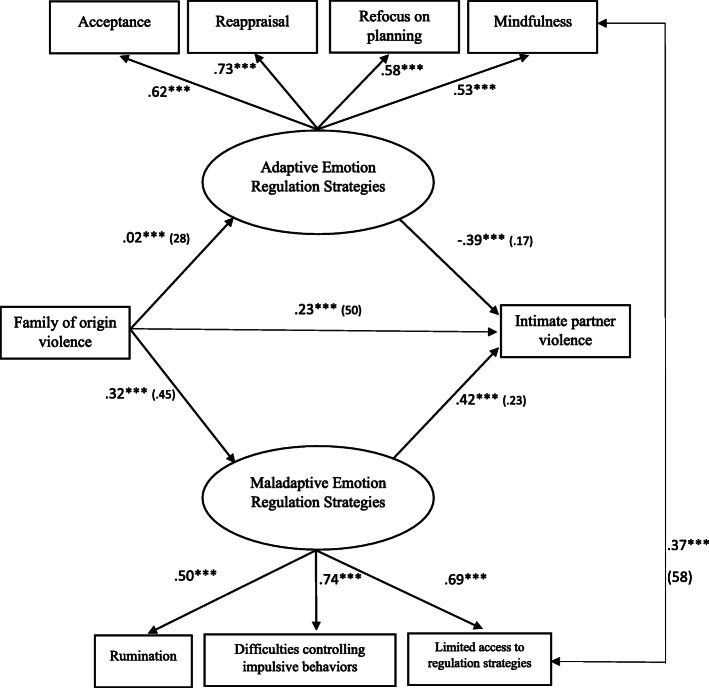
Final structural model for women. Mediating effects of emotion regulation strategies on the relation between family of origin violence and intimate partner violence. Values on paths correspond to the standardized regression coefficients. Standard errors are presented in parenthesis. The maladaptive emotion regulation strategies were a significant mediator. *** *p* < .001

Based on the level of significance of the parameters obtained, it was decided to re-specify the model by omitting the variable of Interference in goal-directed behaviors. As a result, the fit of the model was improved. Specifically for women, the model showed an adequate fit *X*^2^(11, *N* = 838) = 22.75, *p* = .288, GFI = .95, AGFI = .91, NFI = .98, IFC = .97, RMSEA = .05. Therefore, the hypothesized theoretical model for women suggested that family of origin violence predicts intimate partner violence via only maladaptive emotion regulation strategies, thus showing a direct impact. Figure 2 shows standardized path coefficients, illustrating the association between family of origin violence, emotion regulation strategies, and intimate partner violence. The results revealed that only maladaptive emotion regulation strategies mediated the correlation between family of origin violence and intimate partner violence. Thus, high rates of family of origin violence were significant predictors of higher levels of maladaptive strategies, which, in turn, were a significant predictor of higher rates of intimate partner violence.

**Fig. 3:**
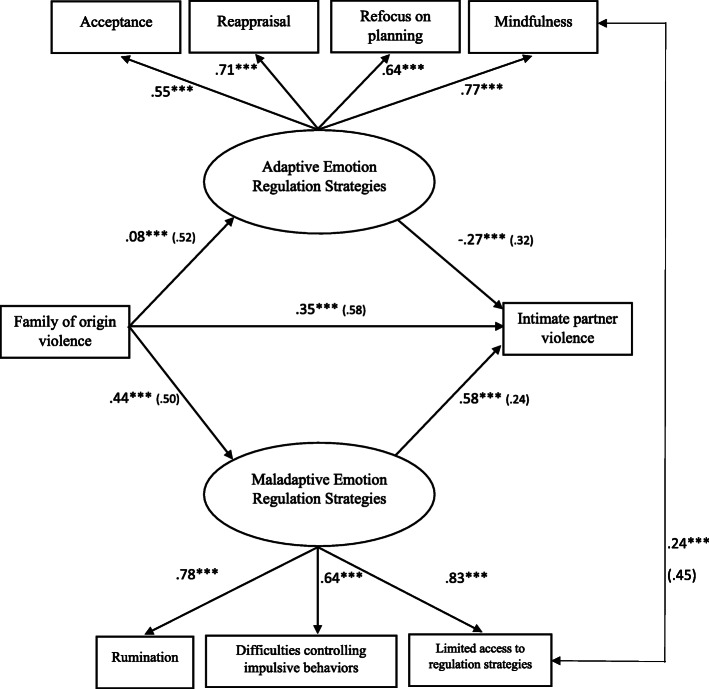
Final structural model for men. Mediating effects of emotion regulation strategies on the relation between family of origin violence and intimate partner violence. Values on paths correspond to the standardized regression coefficients. Standard errors are presented in parenthesis. The maladaptive emotion regulation strategies were a significant mediator. ***p < .001

Meanwhile, the adjustment rates for the hypothesized theoretical model of men were adequate, *X*^2^(11, *N* = 838) = 28.20, *p* = .348, GFI = .97, AGFI = .93, NFI = .97, CFI = .95, RMSEA = .04. The results indicated that only maladaptive emotion regulation strategies mediated the correlation between family of origin abuse and intimate partner violence. Figure 3 shows how all of the direct effects of family of origin violence and of adaptive and maladaptive emotion regulation strategies were significant, except the direct effect of family of origin violence on adequate strategies, which was not significant. In turn, family of origin violence had indirect and significant effects on intimate partner violence via inadequate emotion regulation strategies.

## Discussion

Globally, the correlation between FOV and IPV has been widely documented. However, no model had been designed to explain the association between FOV, emotion regulation strategies, and IPV in the Mexican population. To fill this gap in the scientific literature, this research was aimed to determine whether adaptive or maladaptive emotion regulation strategies mediated the correlation between FOV and IPV in the Mexican population. Similarly, the association between emotion regulation strategies and physical, psychological, and sexual partner violence in women and men from a non-clinical sample was analyzed.

After reviewing the scientific literature, we found that very little research has been carried out to examine the association between family of origin, emotion regulation strategies, and intimate partner violence. One of them is a study conducted in the United States by Bomar (Bomar, 2017), aimed at examining emotion regulation as a moderator of the correlation between FOV and psychological and physical aggression against women. This research included the participation of men who had been arrested for committing various acts of violence against their partners. The results showed that, although no moderating effect was found, exposure to FOV was positively correlated to psychological violence perpetrated only by men who reported low levels of emotion regulation. Likewise, another study including a sample of American university students explored the mediation effect of emotion regulation in the relation between IPV and FOV. Most of participants in this study described that they were dating but living apart (87.1%). In contrast to the present study in which we included different adaptive and maladaptive strategies to evaluate the participants’ emotion regulation; in this other study, researchers measured emotion regulation as a whole construct. Similar to our research, results showed that FOV perpetrated by both parents was associated with emotion regulation difficulties and with IPV when it was reported by sons. In the case of daughters, there was a relation between IPV, emotion regulation difficulties, and FOV when it was rated by their mothers only (Oliveros & Coleman, 2019). Specifically, in Mexico, no studies have been conducted to analyze the effects of the same variables.

In this research, the conducted analyses revealed that FOV correlates to IPV. This association had already been identified frequently in other studies conducted in Mexico and other countries (e.g., Frías & Agoff, 2015; Haj-Yahia, Sousa, & Lugassi, 2019; López-Ramos & Frias, López-Ramos & Frías, 2020; Ludermir, Araújo, Valongueiro, Muniz, & Silva, 2017). In the current study, one of the main findings was that this correlation is mediated by emotion regulation strategies. In the absence of studies that have documented the mediating effect of emotion regulation strategies in the correlation between these two types of violence, we are providing new evidence of the direct and indirect effects we found in the model we are proposing.

Concerning the impact of FOV on emotion regulation strategies, the data obtained showed that, in both models, FOV only had direct effects on maladaptive emotion regulation strategies. This patter has been identified previously in other studies. For instance, children exposed to FOV reported to be unable to regulate negative emotions presenting difficulties to be aware of their emotions. As a result of their lack of awareness, it was more complicated that children had the capacity to distinguish the emotion they were experiencing, have more trouble to identify the origin of their emotions, and were less likely to describe the physical and cognitive sensations that caused the emotions they experienced (Katz, Hessler, & Annest, 2007). Likewise, the conclusion of a meta-analysis examining the association between exposure to FOV and children’s trauma, externalizing and internalizing symptoms was that children who lived exposed to FOV are more prone to express emotional deficits during late stages in their development (Evans, Davies, & DiLillo, 2008).

Based on the results obtained in our study, it was possible to observe that FOV generates in perpetrators a series of dysfunctions that prevents them from adequately control their emotions. Having lived in a family environment characterized by conflicts in the couple and, in many cases, having been abused by their own parents, generates the appropriate conditions for aggressors to develop, since their early childhood, a series of deficiencies in the handling of their emotions. If we add to this that their parents never, or very rarely, taught them to regulate emotions, perpetrators are guided only by the response patterns observed in their family of origin. As the results of this study show, it is not exactly that perpetrators are incapable of accepting, reappraising, focusing or being aware of their own emotions, but rather that they use a series of maladaptive regulatory strategies when trying to control the emotions generated in their romantic relationships. In this way, the conflictive environment experienced during many years in the family of origin ends up producing serious difficulties in the control of negative emotions, involuntary and repetitive thoughts about what was experienced during those years, as well as limitations in identifying the most adequate strategies to regulate emotions.

Regarding the trajectory from maladaptive emotion regulation strategies to partner violence, several studies have documented the correlation between the state of emotion dysregulation and this type of violence. In particular, it has been identified how lack of control in anger management correlated with different types of violence (e.g., Cohn, Jakupcak, Seibert, Hildebrandt, & Zeichner, 2010; Norstrom & Pape, 2010; Sullivan, Helms, Kliewer, & Goodman, 2010). Also, other studies conducted with college students identified that the prevalence of psychological violence increases in couples with larger numbers of deficiencies in their emotion management (Harper, Austin, Cercone, & Arias, 2005). The same trend was found in another study conducted by Gratz, Paulson, Jakupcak, and Tull (2009), in which men who committed more acts of physical violence reported the highest scores on the Difficulties in Emotion Regulation Scale. These authors suggested that, unlike what happens in women, acts of physical violence perpetrated by men toward women are more influenced by the difficulties they present in controlling their emotions. Similarly, Tager, Good, and Brammer (2010) found that men with higher levels of emotion dysregulation were more likely to commit acts of violence against their romantic partners.

In addition to analyzing whether emotion regulation strategies mediated the association between FOV and IPV, it was hypothesized that men have more difficulty controlling their emotions than women. Previous studies had showed discrepancies in their results. In a research conducted by Shorey, Brasfield, Febres, and Stuart (2011), few differences were found between women and men with respect to emotion regulation strategies associated with partner violence. However, in the majority of studies, there are important variations. For instance, in a general population sample, results showed significant differences in the use of emotion regulation strategies. Particularly, women reported higher levels of rumination, positive refocusing, and catastrophizing in comparison to men (Garnefski, Teerds, Kraaij, Legerstee, & van Den Kommer, 2004). In an integrative review of research on the effect of gender on emotion regulation strategies, Nolen-Hoeksema (2012) identified that women tend to use more reappraisal, acceptance, rumination, distraction, problem-solving, and almost all other types of emotion regulation strategies in comparison to men. This trend may have an origin during childhood where studies have found that girls report higher levels of effortful control than boys. In line with these studies, in the current research, we identified significant discrepancies in the management of emotions. While women employ more adaptive emotion regulation, men showed a more definite tendency to use maladaptive emotion regulation. Thus, it became clear that men have more limited access to emotion regulation strategies, are unable to carry out actions aimed at achieving personal goals when they experience negative emotions, and have serious difficulties in controlling their impulses.

Finally, we identified significant discrepancies not only in the management of emotions, but also in the prevalence of violence. The results clearly showed that men reported higher levels of aggression against women, except in terms of psychological violence, where very similar scores were found. This finding reveals that, in Mexican society, men continue to be the main perpetrators of physical, severe physical, and sexual violence against their partners. Similarly, men reported witnessing a greater number of episodes of physical, psychological, and sexual violence between their parents.

Limitations of this study include the retrospective reporting of violence in the family of origin. It is possible that, being unable to exactly recall conflicts between their parents, some participants may have over- or under-reported the different types of violence committed, which may provide less accurate information. At the same time, self-reporting might have led some participants to provide incorrect reports of the episodes of violence they have perpetrated against their romantic partner. It takes truthfulness and honesty on the part of the participants to sincerely acknowledge they have been violent with their partner. Therefore, perpetration rates would vary depending on each participant’s openness. A third limitation of this research relates to the geographic area where data was collected. All participants come from urban and rural areas in Mexico’s central region; in consequence, generalizing the results to a more diverse population is not possible. Finally, family of origin violence only had impact on maladaptive emotion regulation strategies, but we ignore the effect on adaptive strategies. Therefore, future research should replicate these results by including additional instruments measuring other adaptive regulation strategies (e.g., conflict resolution, coping, stress reduction, putting things into perspective) in order to identify the possible effect of family of origin violence on them.

## Conclusions

In sum, we designed a pioneer research with the purpose of examining whether different types of emotion regulation strategies, both adaptive and maladaptive mediate the relationship between family of origin violence and intimate partner violence in the Mexican population. Besides presenting a new theoretical model, the most important strengths of this research highlight that maladaptive emotion regulations strategies are the main predictors in the model for women and men. Therefore, the indirect effects of family of-origin violence were significantly related to intimate partner violence via maladaptive emotion regulation strategies. A second strength points out that although adaptive emotion regulation strategies have a significant effect on intimate partner violence, it appears that maladaptive emotion regulation strategies are an essential factor to understand the prevalence of intimate partner violence. Based on this finding, it is possible to design comprehensive programs to help victims and their families.

A third strength of this study is related to the sex of participants. Mainly in men, maladaptive strategies have a decisive impact in perpetrators’ behavior. When men have developed only this type of strategies or their control of negative emotions is deficient, they are more prone to abuse their partner. These findings can explain the higher prevalence of intimate partner violence committed by Mexican men. Thus, we are contributing to the knowledge of emotion regulation and intimate partner violence providing evidence of the direct and indirect effects found in both models. In addition, regarding family of origin violence, men reported witnessing a greater number of episodes of physical, psychological, and sexual violence between their parents. These findings suggest that since their childhood, men began to witness the presence of conflicts between their parents or in other cases, they were abuse by their own parents. Thus, the abuse experienced during infancy could have severe consequences in their capacity to manager their emotions explaining why men would report higher levels of maladaptive emotion regulation strategies. Following the sequence, the effects on the development of maladaptive strategies are a key factor to explain the high prevalence of intimate partner violence perpetrated by men against women.

On the other hand, regarding the weaknesses and limitations of this research, the first weakness of this study was related to the sociodemographic characteristics of participants. We only collected data in a city in the center of Mexico. Although the population of this city includes millions of people, it does not reflect the social conditions present in other regions of Mexico. In addition, education level can be considered another weakness. Most participants had finished high school; however, only very few participants had a graduate degree. Consequently, it was no possible to identify if participants with a graduate degree are able to manage their emotions using more adaptive emotion regulation strategies. Similarly, the lack of information about history of romantic relationships can be another weakness. Although we collected information regarding family of origin violence, it is necessary to know previous episodes of violence perpetrated particularly during dating relationships. Therefore, it is possible to analyze how victims of violence can control their emotions before they decide to marry/cohabit.

Finally, because this is the first study carried out in Mexico including a structural equation modeling examining the interactions between family of origin violence, emotion regulation strategies, and intimate partner violence, future studies are needed in other Mexican social and geographical context to confirm the results we found in this research. In addition, another suggestion for future studies is the inclusion of more participants with a graduate degree. It will be very interesting to identify if participants with a high education level are more prone to use adaptive strategies in comparison with the education level of participants who use more maladaptive strategies. Furthermore, it is important to design more longitudinal studies. The results found in this first transversal research must be confirmed with other longitudinal studies. Therefore, it will be possible to determine not only the effects of adaptive and maladaptive strategies in the prevalence of intimate partner violence, but also how perpetrators control their emotions over the years. Similarly, future studies should include more emotion regulation strategies. Besides the eight emotion regulations strategies we selected, there are other strategies (e.g., avoidance, direct request, distraction, suppression, and coping) that victims of violence use to manage their emotions. Consequently, the purpose of future studies should be to include more strategies in order to compare the effects of each strategy.

## Data Availability

This study’ s data are available upon request made to the primary author.

## Abbreviations

IPV: Intimate partner violence
FOV: Family of origin violence
